# Deep Immunophenotyping of Human Whole Blood by Standardized Multi-parametric Flow Cytometry Analyses

**DOI:** 10.1007/s43657-022-00092-9

**Published:** 2023-02-23

**Authors:** Jian Gao, Yali Luo, Helian Li, Yiran Zhao, Jialin Zhao, Xuling Han, Jingxuan Han, Huiqin Lin, Feng Qian

**Affiliations:** 1 State Key Laboratory of Genetic Engineering, Shanghai Public Health Clinical Center, Human Phenome Institute, Zhangjiang Fudan International Innovation Center and School of Life Sciences, Fudan University, Shanghai, 200438 China; 2 Ministry of Education Key Laboratory of Contemporary Anthropology, School of Life Sciences, Fudan University, Shanghai, 200438 China; 3 Institute of Immunophenome, International Human Phenome Institutes (Shanghai), Shanghai, 200433 China

**Keywords:** Deep immunophenotyping, Multi-parametric flow cytometry, Human whole blood, Gating strategies, Standardization

## Abstract

Immunophenotyping is proving crucial to understanding the role of the immune system in health and disease. High-throughput flow cytometry has been used extensively to reveal changes in immune cell composition and function at the single-cell level. Here, we describe six optimized 11-color flow cytometry panels for deep immunophenotyping of human whole blood. A total of 51 surface antibodies, which are readily available and validated, were selected to identify the key immune cell populations and evaluate their functional state in a single assay. The gating strategies for effective flow cytometry data analysis are included in the protocol. To ensure data reproducibility, we provide detailed procedures in three parts, including (1) instrument characterization and detector gain optimization, (2) antibody titration and sample staining, and (3) data acquisition and quality checks. This standardized approach has been applied to a variety of donors for a better understanding of the complexity of the human immune system.

**Supplementary Information:**

The online version contains supplementary material available at 10.1007/s43657-022-00092-9.

## Introduction

The human immune system is a complex network of molecules, cells, and tissues that provide effective host defense. Due to its plasticity, the immune system is highly variable between individuals (Brodin and Davis 2017; Liston et al. 2016, 2021). A major aim of phenomics is to quantitatively measure these multiscale networks. Immunophenotyping is essential for analyzing the components and functions of the immune system. The standardized deep immunophenotyping approach will provide an opportunity for longitudinal monitoring of human immune status (Hartmann et al. 2019).

Multi-parametric flow cytometry is a rapidly developing technology that stands as one of the most important analytical tools in the field of immunology (Delmonte and Fleisher 2019). It allows the simultaneous identification and quantification of distinct immune cell subsets at a single-cell level. New antibodies, new fluorochromes, and high-performing flow cytometers are expanding the possibilities for the identification and phenotypic characterization of specific cell populations. However, the increased complexity of immunophenotypic approaches requires optimized antibody panels and fully standardized procedures (Maecker et al. 2010; Maecker et al. 2012).

### Selection of Markers and Clones

**Fig. 1:**
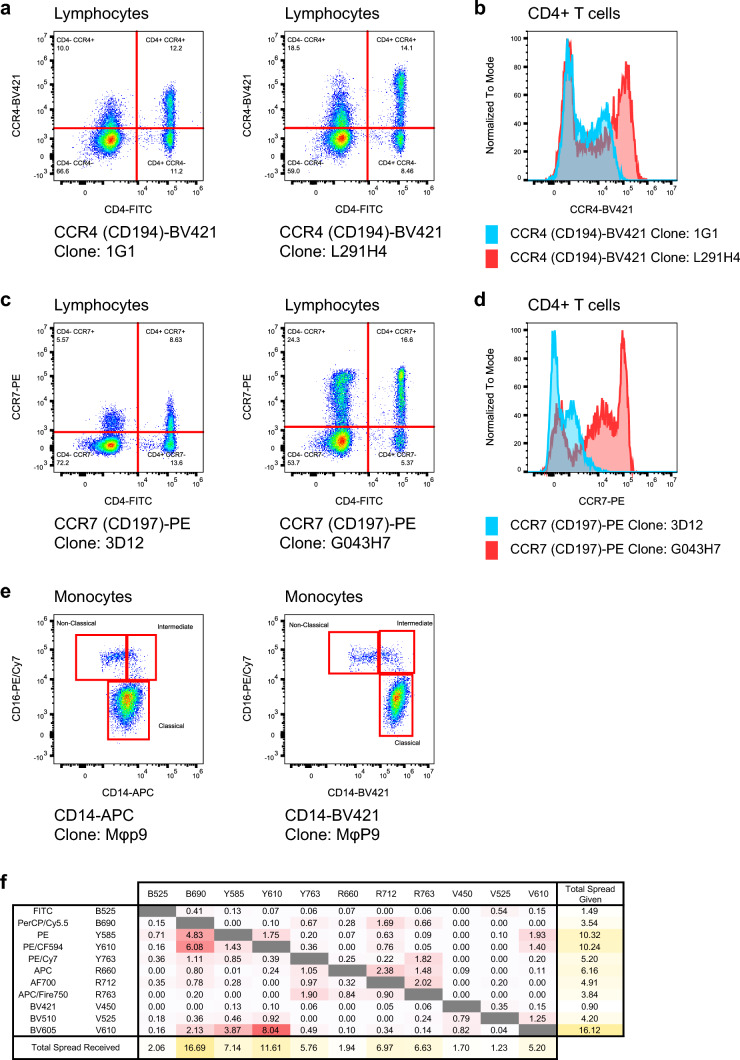
Considerations of antibody clones and fluorochromes during panel development. **a–d** Comparison of different antibody clones for chemokine receptors detection on T cells. Plots showed CCR4 expression (**a, b**) and CCR7 expression (**c, d**) on CD4^+^ cells. The CCR4 dim population and CCR7 dim population were clearly separated from the negative population when the CCR4-clone L291H4 and CCR7-clone G043H7 were used. **e** Brightness of fluorochromes is essential in the discrimination of immune cell subsets. Non-classical monocytes (CD14^low^ CD16^hi^) could be identified when the bright (BV421) fluorochrome was used. **f** A representative spillover spreading matrix from an 11-color configuration of the CytoFLEX LX. The color coding was from no spillover spread (white) to high spillover spread (red). The three fluorophores contributing the least spillover spreading were BV421 (V450), FITC (B525), and PerCP/Cy5.5 (B690). In turn, the three detectors receiving the least spillover spreading are V525, V450, and R660

The design of reproducible antibody panels to produce optimal resolution data for multi-parametric flow cytometry is laborious and time-consuming. As the first step in panel design, it is necessary to clarify the expression level (low to high), expression pattern (bimodal, continuum), and co-expression patterns of the target antigen. Typically, target antigens can be divided into two groups: lineage markers, and function markers. The lineage markers often have known expression patterns, which are used to delineate the target immune cell populations. The function markers are related to the process of cell biology such as cell proliferation, differentiation, activation and exhaustion with unknown expression patterns. An important issue for consideration is the selection of antibody clones. Different antibody clones against the same target antigen have distinct staining patterns from one another (Fig. 1a–d). There are several resources available to help choose commonly used, validated antibody clones such as the series of optimized multicolor immunofluorescence panels (OMIPs) publications (Wang and Creusot 2021) and the OMIP database (https://www.omipcollection.com).

### Choice of Fluorochromes

Fluorochrome selection is an essential step in designing multicolor immunofluorescence panels. Different fluorochromes display a wide range of brightness scales. In general, bright fluorochromes (e.g., PE, BV421) should be reserved for function markers with low expression level, or unknown expression patterns. They can also be chosen for the continuum markers that require clear discrimination between dimly stained and negative cell populations. For example, BV421-conjugated CD14 antibody stained brightly and provided good separation between intermediate monocytes and non-classical monocytes (Fig. 1e). While dim fluorochromes (i.e., FITC, PerCP/Cy5.5) can be assigned to lineage markers with high expression level such as CD3 and CD45.

### Spillover Spreading Error

Spillover spreading has a critical impact on the quality of high-dimensional fluorescent antibody panels for flow cytometry (Nguyen et al. 2013). The signal from one fluorophore spilling into non-target detectors reduces the sensitivity of detectors. An important strategy to minimize spillover spreading is assigning the weak marker to the channel that receives less spread and assigning the backbone marker to the channel that contributes less spread. Figure 1f shows the spillover spreading matrix (SSM) of all possible combinations in the 11-color space for the CytoFLEX LX flow cytometer (Beckman Coulter).

### Standardized Deep Immunophenotyping Workflow

For deep immunophenotyping of human peripheral whole blood, we developed and validated six different 11-color flow cytometry panels (Table 1). This assay characterizes immune cell subsets that circulate in the peripheral blood including all major immune cells such as neutrophils, eosinophils, basophils, monocytes, dendritic cells (DCs), natural killer (NK) cells, T cells, and B cells. It is suited for capturing thousands of immune cell traits including immune cell subset events, frequency, ratio, morphologic properties, and immune cell-surface protein expression levels.

**Table 1:** Overview of the 6 panels for immunophenotyping of human whole blood

Laser	488 nm		561 nm			638 nm			405 nm		
Filter	525/40	690/50	585/42	610/20	763/43	660/20	712/25	763/43	450/45	525/40	610/20
Fluorochrome	FITC	PerCP/Cy5.5	PE	PE/CF594	PE/Cy7	APC	AF700	APC/Fire750	BV421	BV510	BV605
*Panel 1: PMNs, monocytes and DCs panel*											
Specificity	CD64	CD15	CD86	CD11c	CD123	CD16	HLA-DR	LIN^a^	CD14	CD45	CD38
*Panel 2: NK, NKT, MAIT and γδT cells*											
Specificity	TCR Vα7.2	NKp46	TCR Vδ2	NKG2D	CD16	TCR γδ	CD161	CD3	CD56	CD45	CD8
*Panel 3: T eff/mem and Treg cells panel*											
Specificity	CCR7	CD3	CD95	CD25	HLA-DR	CD39	CD45RA	CD4	CD127	CD45	CD8
*Panel 4: T cells functional status panel*											
Specificity	CD85j	CD3	PD-1	CD69	CD28	CD57	HLA-DR	CD4	CD38	CD45	CD8
*Panel 5: Th, Tc, Tfh and Treg cells panel*											
Specificity	CXCR5	CD3	ICOS	CD25	CXCR3	CCR6	CD127	CD4	CCR4	CD45	CD8
*Panel 6: B cells panel*											
Specificity	CD21	CD20	CD24	CD11c	IgD	CD38	IgG	CD19	CD27	CD45	IgM

FITC, fluorescein isothiocyanate; PE, phycoerythrin; PerCP, peridinin–chlorophyll–protein complex; Cy5.5, cyanine 5.5; Cy7, cyanine 7; APC, allophycocyanin; AF, Alexa Fluor; BV: Brilliant Violet. Eff/mem, effector/memory; LIN, lineage

^a^LIN includes CD3, CD19 and CD56

The multi-step procedure must be standardized for flow cytometric immunophenotyping. These steps involve the instrument characterization (Nguyen et al. 2013; Perfetto et al. 2006, 2012), detector gain determination (Kalina et al. 2012; Maciorowski et al. 2017), antibody titration, sample staining (Berhanu et al. 2003), and data analysis (Monaco et al. 2016). Here, we describe in detail aspects of the procedures that are crucial for the deep immunophenotyping of human whole blood by multiparametric flow cytometry.

## Materials and Methods

### Materials and Reagents

Fresh human peripheral whole blood was collected in heparin-coated tubes or ethylenediaminetetraacetic acid (EDTA) anticoagulant tubes. All fluorochrome-conjugated antibodies used are listed in Supplementary Table S1. Whole blood samples were lysed with FACS™ Lysing Solution (BD, Cat#349202), and washed with phosphate buffered saline (PBS, Wisent, Cat#311-010-CL). After the final wash, cells were resuspended in paraformaldehyde (PFA; Sangon Biotech, Cat#A500684-0500). Eight-peaks Rainbow Calibration Particles (RCPs; Spherotech, Cat#RCP-30-5A), Anti-Mouse Ig, κ/Negative Control Compensation Particles Set (BD, Cat# 552843) were used in the procedures described in this protocol. All samples were acquired on a CytoFLEX LX Flow Cytometer (Beckman Coulter, Cat#C00446) through CytExpert_v2.4 (Beckman Coulter), and analyzed with FlowJo_v10.8.1 (BD). Data plots and statistical analyses were done in FlowJo_v10.8.1 and Microsoft Excel_2016 (Microsoft Corporation).

### Reagent Setup

- Stain buffer: PBS with 2% fetal bovine serum (FBS; Gibco, Cat#10099141) (vol/vol). Stain buffer can be stored at 2–8 °C for up to 2 weeks. Do not freeze.
- 1 × Lysing Solution: Dilute the 10 × concentrate FACS™ Lysing Solution 1:10 with ddH_2_O at room temperature (RT, 20–25 °C).
- 1% PFA: Dilute the 4% PFA (Sangon Biotech, Cat#A500684-0500) 1:4 with PBS.
- Antibody premixes: Mix the correct concentration of the antibodies, as determined by titration experiments, to 20 µL of stain buffer per sample (chemokine receptor antibodies premix and surface-staining antibodies premix). Centrifuge before use at 14,000 × *g* for 2 min at RT to remove antibodies aggregates.

## Procedure

### Instrument Characterization and Detector Gain Optimization

#### Calibration of Detector Linearity and Gain Range

1. Vortex the 8-peaks RCPs vigorously. Add 2–3 drops of particles to 1 mL of ddH_2_O in a 5 mL 12 × 75 mm polypropylene tube.
2. Check CytoFLEX LX flow cytometer's lasers, mirrors, and filters. Complete daily instrument-specific start-up and quality control (QC) procedures.
3. Adjust the Forward scatter (FSC)/Side scatter (SSC) gain to place the beads on a scale in the light scatter plot and set a gate around the singlet cell population on the FSC-area (FSC-A) vs FSC-height (FSC-H) dot plot to exclude aggregates.
4. Set gains for all detectors to 25 V (Except FSC/SSC channel).
5. Run 8-peaks RCPs and negative control compensation particles (CCPs), respectively. Acquire 5000 events for each kind of particle.
6. Increase gain by 50 V for each detector and repeat Step 5.
7. Export data in FCS3.1 file format and load into FlowJo.
8. Calculate the median fluorescence intensity (MFI) of both the second brightest bead peak (referred to as P7_MFI) and the brightest bead peak (referred to as P8_MFI). Calculate the detector linearity as follows: ${\text{linearity}} = \frac{P8 \_MFI - P7\_MFI}{{P7\_MFI}}$ (Fig. S1a).
9. Calculate the robust coefficient of variance (rCV) for the second brightest bead peak (referred to as P7_rCV).
10. Calculate the MFI of the negative CCPs and defined them as background (referred to as B_MFI). Calculate the signal-to-background ratio (SBR) as follows: $\mathrm{SBR}=\frac{P7\_MFI}{B\_MFI}$ (Fig. S1a).
11. For each detector, plot the detector linearity, P7_rCV, and SBR on one graphic. Here the detector gain range can be defined as the gain point showing the highest SBR co-occurring with the lowest rCV and a slope of zero on the linearity curve (Fig. S1b).
12. Repeat this procedure when a new laser, detector, or filter is installed.

#### Calculation of Minimal Detector Gain

13. Gate the second dimmest peak of the 8-peaks RCPs and calculate the rCV of that peak (referred to as P2_rCV) in each fluorescence detector for gain ranges as described in the previous section.
14. Generate a plot showing the P2_rCV on the *y*-axis relative to the gain on the *x*-axis. When the slope of the curve is zero, the gain is minimal and brings the negative population out of the detector’s electronic noise range (Fig. S1c).

CRITICAL Gain titration to deliver optimal resolution (see supplementary optional procedure subheading Gain Titration (Voltration) Experiment).

### Antibody Titration and Sample Staining

CRITICAL

1. Human peripheral whole blood should be processed as soon as possible after collection.
2. Human peripheral whole blood should be transported and stored at RT.
3. For biosafety and to prevent sample contamination, whole blood samples should be processed in a biosafety cabinet.

#### Antibody Titration

15. Plan on using 120 μL/tube to stain, and a five-point, twofold dilution series.
16. Prepare five 200 μL cap tubes for each antibody. Label tubes with #1–#5 (4 dilutions and one unstained sample).
17. Transfer 32 μL stain buffer into cap tube #1, and add 8 μL of antibody. Mix thoroughly by a vortex.
18. Add 20 μL of stain buffer into cap tubes #2–#5.
19. Pipette 20 μL of antibody dilution from cap tube #1 into cap tube #2 and mix thoroughly by vortex.
20. Pipette 20 μL of antibody dilution from cap tube #2 into cap tube #3 and mix thoroughly by vortex.
21. Pipette 20 μL of antibody dilution from cap tube #3 into cap tube #4 and mix thoroughly by vortex.
22. Pipette 20 μL of antibody dilution from cap tube #4 and discard into waste.
23. Prepare five 5 mL 12 × 75 mm polypropylene tubes for each antibody. Label tubes with #1–#5 (4 dilutions and one unstained sample). And transfer 100 μL of whole blood into each polypropylene tube.
24. Transfer each antibody dilution into the corresponding 12 × 75 mm polypropylene tube. Mix thoroughly by a vortex. Thus, the antibody final concentrations are 1:30, 1:60, 1:120, 1:240, and 0, respectively.
25. For chemokine receptor antibodies, incubate for 15 min in a 37 °C water bath in the dark. For remaining surface-staining antibodies, incubate for 15 min at RT in the dark.
26. Add 2 mL 1 × Lysing Solution to each labelled 5 mL tube.
27. Mix thoroughly by vortex, and incubate for 15 min at RT in the dark.
28. Centrifuge the cells at 500 × *g* for 5 min at RT.
29. Flick or aspirate to remove supernatant and wash cells with 2 mL PBS at 500 × *g* for 5 min at RT.
30. Flick or aspirate to remove supernatant and wash twice.
31. Flick or aspirate to remove supernatant and resuspend in a final volume of 250 μL 1% PFA. Store at 2–8 °C and protect from light until acquisition. Do not freeze.
32. Check CytoFLEX LX flow cytometer's lasers, mirrors, and filters. Complete daily instrument-specific start-up and QC procedures.
33. Load the dilution series samples on the flow cytometer.
34. Adjust the FSC/SSC gain to place the cells on a scale in the light scatter plot and set a gate around the singlet cell population on the FSC-A vs FSC-H dot plot to exclude aggregates.
35. Adjust the detector gain determined with the previous procedure (see subheading Calculation of Minimal Detector Gain), and acquire 200 μL per sample.
36. Export data in FCS3.1 file format and load into FlowJo.
37. Calculate the stain index (SI) for each sample using the following formula: $\mathrm{SI}=\frac{MFI(pos) - MFI(neg)}{2 \times rSD(neg)}$.
38. Generate a plot showing the SI on the *y*-axis relative to the dilution on the *x*-axis. The dilution that represents the best SI with the lowest concentration of antibody is the dilution to use (Fig. S2).

#### Compensation Setup Experiment

39. Vortex the BD CompBeads vigorously. In the meantime, label 5 mL 12 × 75 mm polypropylene tubes for each fluorochrome-conjugated antibody.
40. Pipette 80 μL of stain buffer into each tube and then add 20 μL of CompBeads (containing 10 µL positive control beads, along with 10 µL negative control beads).
41. Add the correct concentration of the antibodies to each tube, which is determined by the titration experiments (see subheading Antibody Titration).
42. Prepare one additional tube as a negative control (without any antibody).
43. Mix thoroughly by vortex, and incubate for 15 min at RT in the dark.
44. Add 2 mL stain buffer to each tube.
45. Centrifuge the cells at 500 × *g* for 5 min at RT.
46. Flick or aspirate to remove supernatant and resuspend in a final volume of 250 μL stain buffer. Store at 4 °C and protect from light and moisture until acquisition. Do not freeze.
47. Check CytoFLEX LX flow cytometer's lasers, mirrors, and filters. Complete daily instrument-specific start-up and QC procedures.
48. Create a new compensation experiment, and select the channel requiring compensation calculation and the sample type.
49. Adjust the FSC/SSC gain to place the BD CompBeads on a scale in the light scatter plot and set a gate around the singlet cell population on the FSC-A vs FSC-H dot plot to exclude aggregates.
50. Adjust the detector gain determined with the previous procedure (see subheading Calculation of Minimal Detector Gain).
51. Load one of the single-stained samples, and acquire 5000 events per tube.
52. Repeat Step 13 for all single-stained samples.
53. Check all acquired samples, and move the positive or negative gates in the plot to define the target population.
54. Click the “Compensation Calculation” button to generate the compensation matrix.

CRITICAL

1. Carefully check the origin of the antibody. The BD CompBeads Anti-Mouse Ig, *κ* particles, which bind mouse *κ* light chain-bearing immunoglobulin. Vendors such as Invitrogen and BioLegend provide compensation beads that can bind with antibodies of mouse, rat, hamster, rabbit, and human origin.
2. If compensation beads are not available at the time that can bind antibodies of rat or hamster origin, the original antibody can be replaced with an antibody of the same fluorochrome.
3. Routinely performing the compensation setup experiment.
4. Characterization of spillover spreading error (SE) to reveal fluorescence spectrum interactions (see supplementary optional procedure subheading Spillover Spreading Matrix Determination Experiment).

#### Whole Blood Staining Procedures

55. Prepare 5 mL 12 × 75 mm polypropylene tubes for each panel, and label tubes with the name of the panel.
56. Transfer 100 μL of whole blood into each labelled 5 mL tube.
57. Spin shortly (about 20 s) all antibody premixes.
58. For panel 1, 2, 3 and 4, proceed to procedures 59–66, for panel 5, skip procedures 59–66 and proceed to procedures 67–76, and for panel 6, skip procedures 59–76 and proceed to procedures 77–86.

#### For Panel 1, 2, 3 and 4

59. Add each antibody premix to the corresponding tube.
60. Mix thoroughly by vortex, and incubate for 15 min at RT in the dark.
61. Add 2 mL 1 × Lysing Solution to each labelled 5 mL tube.
62. Mix thoroughly by vortex, and incubate for 15 min at RT in the dark.
63. Centrifuge the cells at 500 × *g* for 5 min at RT.
64. Flick or aspirate to remove supernatant and wash cells with 2 mL PBS at 500 × *g* for 5 min at RT.
65. Flick or aspirate to remove supernatant and wash twice.
66. Flick or aspirate to remove supernatant and resuspend in a final volume of 250 μL 1% PFA. Store at 2–8 °C and protect from light until acquisition. Do not freeze.

#### For Panel 5

67. Add chemokine receptor antibodies premix in the corresponding tube.
68. Mix thoroughly by vortex, and incubate for 15 min in a 37 °C water bath in the dark.
69. Add remaining surface-staining antibodies premix in the corresponding tube.
70. Mix thoroughly by vortex, and incubate for 15 min at RT in the dark.
71. Add 2 mL 1 × Lysing Solution to the corresponding tube.
72. Mix thoroughly by vortex, and incubate for 15 min at RT in the dark.
73. Centrifuge the cells at 500 × *g* for 5 min at RT.
74. Flick or aspirate to remove supernatant and wash cells with 2 mL PBS at 500 × *g* for 5 min at RT.
75. Flick or aspirate to remove supernatant and wash twice.
76. Flick or aspirate to remove supernatant and resuspend in a final volume of 250 μL 1% PFA. Store at 2–8 °C and protect from light until acquisition. Do not freeze.

CRITICAL If necessary, prepare three times 100 µL of whole blood stained in parallel and pooled directly before measurement to obtain sufficient cell numbers.

#### For Panel 6

77. Add 2 mL 1 × Lysing Solution to the corresponding tube.
78. Mix thoroughly by vortex, and incubate for 15 min at RT in the dark.
79. Centrifuge the cells at 500 × *g* for 5 min at RT.
80. Flick or aspirate to remove supernatant and wash cells with 2 mL PBS at 500 × *g* for 5 min at RT.
81. Flick or aspirate to remove supernatant and resuspend in 100 μL stain buffer.
82. Add Panel-6 antibodies premix in the corresponding tube.
83. Mix thoroughly by vortex, and incubate for 15 min at RT in the dark.
84. Add 2 mL PBS to the corresponding tube, and mix thoroughly by the vortex.
85. Centrifuge the cells at 500 × *g* for 5 min at RT.
86. Flick or aspirate to remove supernatant and resuspend in a final volume of 250 μL 1% PFA. Store at 2–8 °C and protect from light until acquisition. Do not freeze.

CRITICAL If necessary, prepare three times 100 µL of whole blood stained in parallel and pooled directly before measurement to obtain sufficient cell numbers.

### Data Acquisition and Quality Checks

#### Data Acquisition

87. Check CytoFLEX LX flow cytometer's lasers, mirrors, and filters. Complete daily instrument-specific start-up and QC procedures.
88. Before the acquisition, lasers should warm up for 15 min.
89. Adjust the FSC/SSC gain to place the cells on a scale in the light scatter plot and set a gate around the singlet cell population on the FSC-A vs FSC-H dot plot to exclude aggregates.
90. Adjust the detector gain determined with the previous procedure (see subheading Calculation of Minimal Detector Gain).
91. Acquire samples (200 μL per panel per sample) using CytExpert at a high flow rate (60 μL/min). And it is recommended to acquire more than 200,000 events per sample.

#### Compensation Quality Check

92. Load FCS3.1 files into CytExpert, which is software that controls the CytoFLEX LX flow cytometer operation.
93. Apply the compensation matrix generated with the previous procedure (see subheading Preparation of compensation controls).
94. Create N-by-N bivariate plots of all combinations to check the current sample's fluorescent parameters compensation.
95. When the compensation matrix is not suitable, check the following three items: (1) the single-stained compensation controls should be matched to the correct detectors; (2) positive and negative populations should be correctly determined by the gates in each control; (3) no contamination occurred during the preparation of the single-stained samples.
96. Further manual adjustment of the compensation is required if any of the compensation is not proper.

#### Data Cleaning

97. Install the “FlowAI” Plugin in FlowJo (see the Installing Plugins page for details: https://docs.flowjo.com/flowjo/plugins-2/installing-plugins/).
98. Load the FCS3.1 files into FlowJo which have been checked the compensation.
99. Access and initiate FlowAI via the Plugins Menu in FlowJo (Workspace Tab— > Populations Band— > Plugins menu) to remove any anomalies. And select cleaned data for the next step of data analysis.
100. Set a gate around the singlet cell population on the FSC-A vs FSC-H dot plot and FSC-width (FSC-W) vs FSC-A dot plot to exclude aggregates.
101. Clean up the data based on the number of immune cell subsets after manual gating by setting the limits of detection (LOD) to > 20 cells and the lower limit of quantification (LLOQ) to > 50 cells. A population of cells with fewer than 50 cells in 80% of samples is filtered out.

### Troubleshooting

**Table:** 

Problem	Possible reason	Potential solution
rCVs on either the 8-peaks RCPs or negative CCPs are greater in one detector than another detector	Dichroic steering mirror is out of alignment	Adjust the steering mirror alignment
Detector’s linearity curve does not reach a plateau	Detector is faulty or poorly aligned	Replace the detector
rCVs on the 8-peaks RCPs increase with gain setting series	Detector is faulty	Replace the detector
SI curve does not reach a plateau in the voltration experiment	Voltration process does not reach a maximal resolution	Continue the voltration process at higher gain setting series
Positive compensation beads with the specific fluorochrome-conjugated antibody are negative	The antibody is incompatible with the bead	Species-specific antibody compensation beads should be used
Poorly discriminate the positive population from the negative population	Antibodies were not used at optimal concentrations, or staining method was not optimized for chemokine receptor antibodies	Titrate all antibodies before experiments, or stain with chemokine receptor antibodies at 37 °C
Many combinations are undercompensated or overcompensated	The signal of the positive compensation control was not as bright as the whole blood sample	Use whole blood samples for the compensation control
Interruptions during acquisition (visualized via the time parameter)	Instability of flow stream	Resuspend cells to exclude aggregates and exclude the events with flowAI

## Anticipated Results

### Polymorphonuclear Leukocytes (PMNs), Monocytes, and DCs

**Fig. 2:**
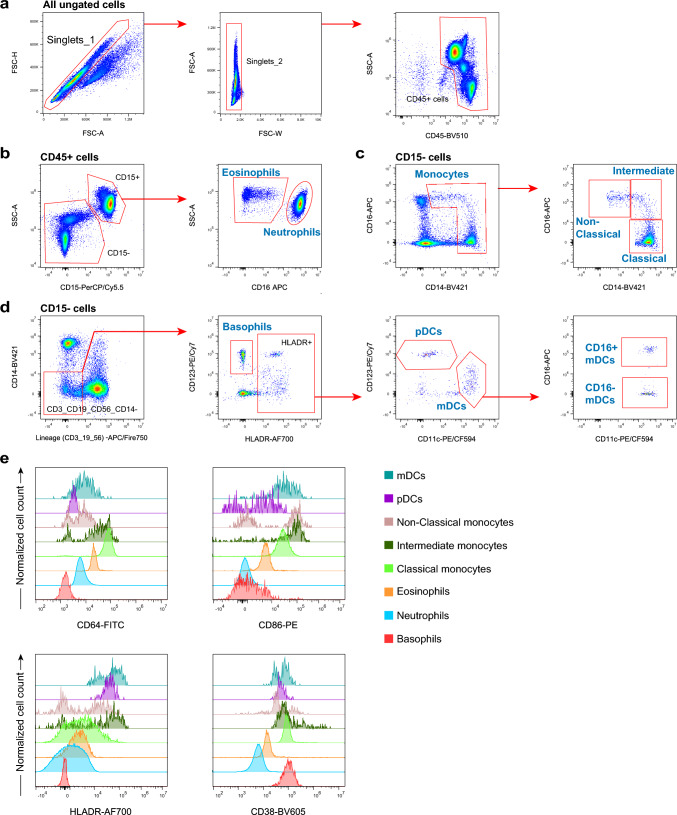
Gating strategies for the PMNs, Monocytes and DCs panel (Panel 1). **a** CD45^+^ cells were identified by their CD45 expression after exclusion of doublets by cross-checking the forward scatter (FSC) signal for its area (A) versus height (H) and width (W) characteristics. **b** CD45^+^ leukocytes were distinguished into CD15^+^ and CD15^−^ populations, then neutrophils and eosinophils were identified by their CD16 expression on CD15^+^ cells. **c** Identification of monocytes. Total monocytes were separated on an CD14 versus CD16 dot plot from CD15^−^ SSC^low^ cells. Monocytes were identified and further distinguished into classical monocytes (CD14^hi^ CD16^−^), intermediate monocytes (CD14^hi^ CD16^+^) and non-classical monocytes (CD14^+^ CD16^hi^) by their CD14 and CD16 expression. **d** Identification of basophils and dendritic cells. Lineage negative cells were identified by their expression of CD3, CD14, CD19 and CD56. Then the basophils (HLA-DR^−^ CD123^+^) were gated on an anti-HLA-DR versus anti-CD123 dot plot. mDCs (CD11c^+^ CD123^−^) and pDC (CD11c^−^ CD123^+^) were identified by their differential CD11c and CD123 expression. The mDCs were further subdivided into CD16^+^ mDC and CD16^−^ mDC by their CD16 expression. **e** Expression of functional markers (CD69, CD28, HLA-DR, and CD38) by different PMNs, monocytes, and DCs subsets. A reference population of CD3^−^ CD56^−^ cells from the same sample was served as a negative expression control. Basophils can be used as a negative expression control for CD64, CD86, and HLA-DR

To characterize major subsets of innate immune cells, CD45^+^ cells were identified after the exclusion of doublets (Fig. 2a). We include the marker CD16 to differentiate eosinophils from neutrophils among the CD15^+^ population (Fig. 2b) (Gustafson et al. 2015; Patin et al. 2018; Ruhle et al. 2016). The basophils were identified as HLA-DR^−^ CD123^+^ cells on lineage (CD3/CD14/CD19/CD56) negative population. The lineage^−^ HLA-DR^+^ population was further distinguished into myeloid dendritic cells (mDCs) and plasmacytoid dendritic cells (pDCs) according to their differential expression of CD11c and CD123. (Fig. 2d) (Mair and Prlic 2018; Park et al. 2020). Monocytes are early responders to pathogens in acute infections. The monocytes were discriminate different inflammatory/differentiation stages by their CD14 and CD16 expression: classical monocytes (CD14^hi^ CD16^−^), intermediate monocytes (CD14^hi^ CD16^+^) and non-classical monocytes (CD14^+^ CD16^hi^) (Fig. 2c) (Bocsi et al. 2014; Hally et al. 2022; Park et al. 2020; Staser et al. 2018). The expressions of CD64, CD86, CD38, and HLA-DR can be evaluated to determine the activation state of the PMNs, monocytes, and DCs (Fig. 2e).

#### Unconventional T Cells and NK Cells

**Fig. 3:**
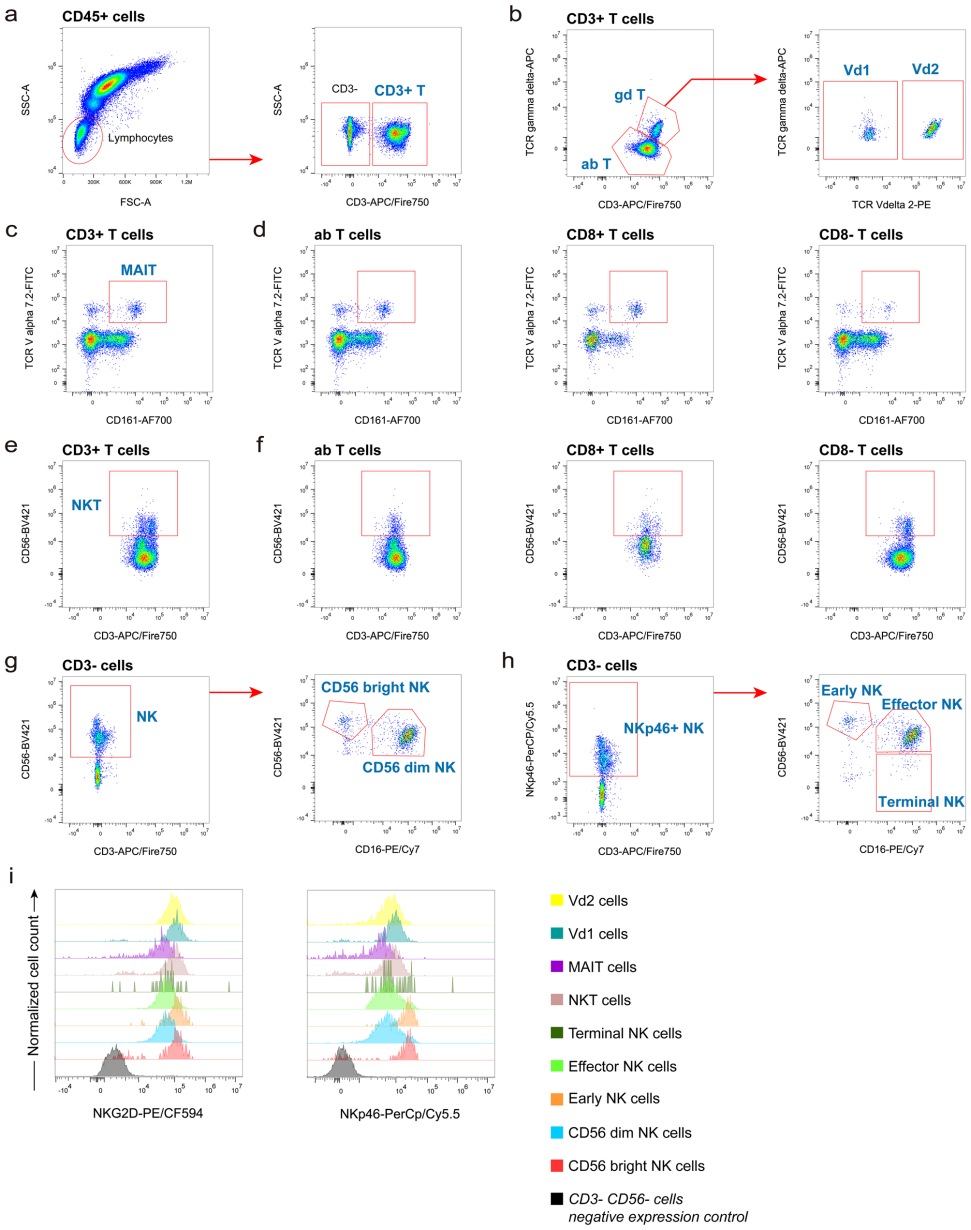
Gating strategies for the NK, NKT, MAIT and γδ T cells panel (Panel 2). Exclusion of doublets and gating of CD45^+^ cells as shown for panel 1 (Fig. 2a). **a** Lymphocytes were gated on the FSC-A versus SSC-A dot plot, and T cells were identified by their CD3 expression. **b** Identification of αβ T cells and γδ T cells (anti-CD3 versus anti-TCR γδ plot). The γδ T cells were further subdivided into Vδ1 cells and Vδ2 cells (anti-TCR Vδ2 versus anti-TCR γδ plot). **c** Identification of MAIT cells (anti-CD161 versus anti-TCR Vα7.2 plot) and MAIT cells in αβ cells, CD8^+^ T cells and CD8^−^ T cells (**d**). **e** Identification of NKT cells (anti-CD3 versus anti-CD56 plot) and NKT cells in αβ cells, CD8^+^ T cells and CD8^−^ T cells (**f**). **g**, **h** Two gating strategies to identify NK cells. NK cells can be determined by CD3^−^ CD56^+^ cells (**e**) and subsequently distinguished into two subsets by their CD56 and CD16 co-expression into CD56 bright NK cells (CD56^hi^ CD16^−/low^) and CD56 dim NK cells (CD56^low^ CD16^+^). NK cells can also be determined by CD3^−^ NKp46^+^ cells (**f**), which were divided into early NK cells (CD56^hi^ CD16^−/low^), effector NK cells (CD56^low^ CD16^+^) and terminal NK cells (CD56^−^ CD16^+^). **i** Expression of activating receptors (NKG2D and NKp46) by different cell subsets

The unconventional T cells are a diverse group of relatively rare lymphocytes which play important roles in disease (Bae et al. 2018; Godfrey et al. 2019, 2018, 2016; Mayassi et al. 2021; Petley et al. 2021; Silva-Santos et al. 2019; Wilkinson and Cerrone 2020). Lymphocytes were distinguished into CD3^+^ and CD3^−^ populations (Fig. 3a). We identified γδ T cells and their subsets on CD3^+^ T cells by markers TCR γδ and TCR Vδ2 (Fig. 3b) (Gherardin et al. 2014; Hertoghs et al. 2020; Mahnke et al. 2013c). The mucosal-associated invariant T (MAIT) cells were identified by their CD161^+^ TCR Vα7.2^+^ phenotype in total T cells (Fig. 3c) and αβ cells, CD8^+^ T cells and CD8^−^ T cells (Fig. 3d) (Gherardin et al. 2014; Hertoghs et al. 2020). NK cells have a strong cytolytic function against virus-infected cells. We included CD56 as common NK cell marker to identify NK and NKT cells (Fig. 3e, g) (Costanzo et al. 2015; Eller and Currier 2012; Hertoghs et al. 2020; Mahnke et al. 2013c). NKT cells are further identified in αβ cells, CD8^+^ T cells and CD8^−^ T cells (Fig. 3f). Previous studies have shown the presence of NK cells that do not express CD56, especially in the peripheral blood of patients with chronic viral infections (Bjorkstrom et al. 2010). Therefore, we also provide a gating strategy to identify NK cells as CD3^−^ NKp46^+^ (Fig. 3h) (Park et al. 2020; Patin et al. 2018). Additional two activating receptors (NKG2D and NKp46) distribution among different cell subsets were evaluated (Fig. 3i).

#### T Cell Subsets

**Fig. 4:**
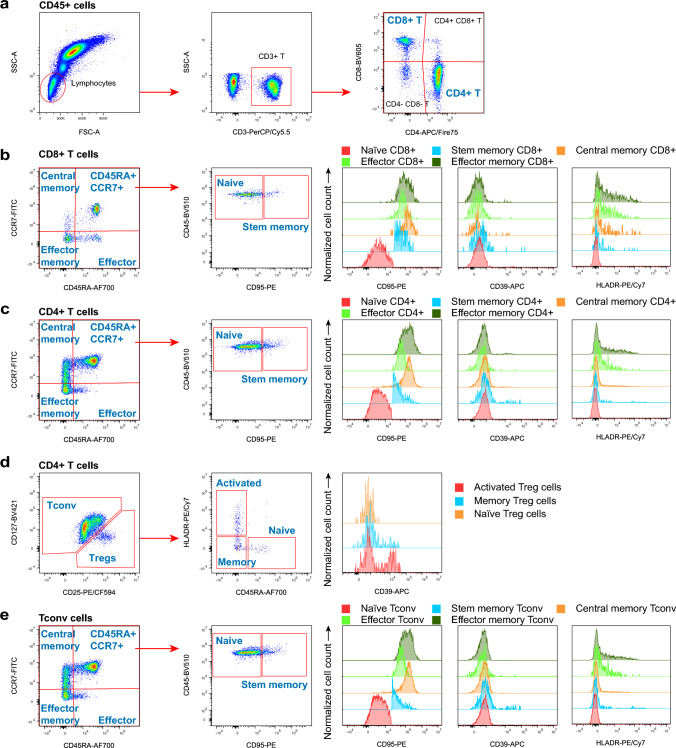
Gating strategies for the T eff/mem and Treg cells panel (Panel 3). Exclusion of doublets and gating of CD45^+^ cells as shown for panel 1 (Fig. 2a). **a** Lymphocytes were gated on the FSC-A versus SSC-A dot plot, and T cells were identified by their CD3 expression. CD4^+^ T cells, the double-negative T cells (CD4^−^ CD8^−^ T cells), CD8^+^ T cells, and double-positive T cells (CD4^+^ CD8^+^ T cells) were identified by their expression of CD4 and CD8. **b**, **c** Identification of different maturation stages of CD8^+^ T cells (**b**) and CD4^+^ T cells (**c**). Naïve T cells (CD45RA^+^ CCR7^+^ CD95^−^), stem memory T cells (CD45RA^+^ CCR7^+^ CD95^+^), effector T cells (CD45RA^+^ CCR7^−^), effector memory T cells (CD45RA^−^ CCR7^−^), and central memory T cells (CD45RA^−^ CCR7^+^) were identified by their expression levels of CD45RA, CCR7 and CD95. The expression levels of surface markers (CD95, CD39, and HLA-DR) of T cells at different stages of maturation were further evaluated. **d** Tconv and Treg cells were identified by their expression of CD25 and CD127. Treg cells were further distinguished into CD45RA^−^ HLA-DR^+^ activated, CD45RA^−^ HLA-DR^−^ memory, and CD45RA^+^ HLA-DR^−^ naïve regulatory T cells. Treg subpopulations were analyzed for the CD39 activation marker expression. **e** Identification of different stages of Tconv cells and analysis of surface markers

T cells are important mediators in cell-mediated immunity, and numerous different T cell subsets were identified and characterized. To identify T cells the pan marker CD3 was included in panels 2, 3, 4, and 5. CD4^+^ T cells, CD8^+^ T cells, the double-negative T cells (CD4^−^ CD8^−^ T cells), and double-positive T cells (CD4^+^ CD8^+^ T cells) were identified by their differential CD4 and CD8 expression (Fig. 4a). The CD8^+^ T cells (Fig. 4b) and CD4^+^ T cells (Fig. 4c) were further distinguished into naïve T cells (CD45RA^+^ CCR7^+^ CD95^−^), stem memory T cells (TSCM, CD45RA^+^ CCR7^+^ CD95^+^), effector T cells (CD45RA^+^ CCR7^−^), effector memory T cells (EM, CD45RA^−^ CCR7^−^), and central memory T cells (CM, CD45RA^−^ CCR7^+^) by their CD45RA, CCR7 and CD95 expression level (Mahnke et al. 2012; Staser et al. 2018). The expression levels of surface markers (CD95, CD39, and HLA-DR) of T cells at different stages of maturation were further evaluated. The regulatory T cells (Tregs) were identified by their CD25^hi^ CD127^−/low^ phenotype on CD4^+^ T cells and the activation status was determined by the markers CD45RA and HLA-DR (Fig. 4d) (Biancotto et al. 2012; Liechti and Roederer 2019b; Mahnke et al. 2013a; Murdoch et al. 2012; Park et al. 2020). And Treg subpopulations were analyzed for the CD39 activation marker expression. In addition, the conventional T (Tconv) cells were identified and further distinguished into different maturation stages in Fig. 4e.

**Fig. 5:**
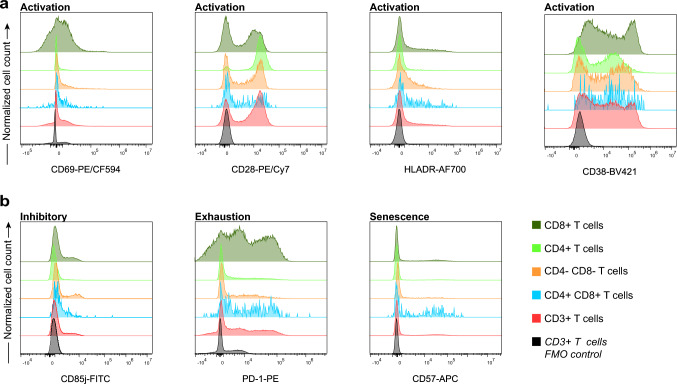
Expression of activation, inhibitory and exhaustion markers by different T cell subsets (T cell functional status panel, Panel 4). Exclusion of doublets and gating of CD45^+^ cells as shown for panel 1 (Fig. 2a). CD4^+^ T cells, CD8^+^ T cells, CD4^−^ CD8^−^ T cells, and CD4^+^ CD8^+^ T cells were identified by their expression of CD4 and CD8 (shown in Fig. 4a). **a** Expression of activation markers (CD69, CD28, HLA-DR, and CD38) by different T cell subsets. **b** Expression of the inhibitory receptor (CD85j), exhaustion marker PD-1, and terminal differentiation marker CD57 by different T cell subsets. Lower histograms represent the fluorescence minus one (FMO) control expression for the lineage-gated CD3^+^ T cells. FMO controls for the functional markers were performed on one whole blood sample

To determine the activation status of T cells and their subsets (CD4^+^ T cells, CD8^+^ T cells, CD4^−^ CD8^−^ T cells, and CD4^+^ CD8^+^ T cells), the expression of activation markers CD69, CD28, HLA-DR, and CD38 was analyzed in Panel 4 (Fig. 5a). The inhibitory receptor (CD85j), exhaustion marker programmed cell death protein 1 (PD-1), and senescence marker CD57 expression was also examined in these T cell subsets (Fig. 5b) (Healy and Murdoch 2016; Swanson and Seder 2020). CD85j^+^ T cells have been shown to be associated with aging (Alpert et al. 2019). The PD-1 is upregulated after T cell activation to prevent an excessive immune response. The CD57 was a marker of terminal differentiation and associated with autoimmune diseases, infectious diseases, and cancer (Characiejus et al. 2008; Palmer et al. 2005; Pedroza-Seres et al. 2007).

**Fig. 6:**
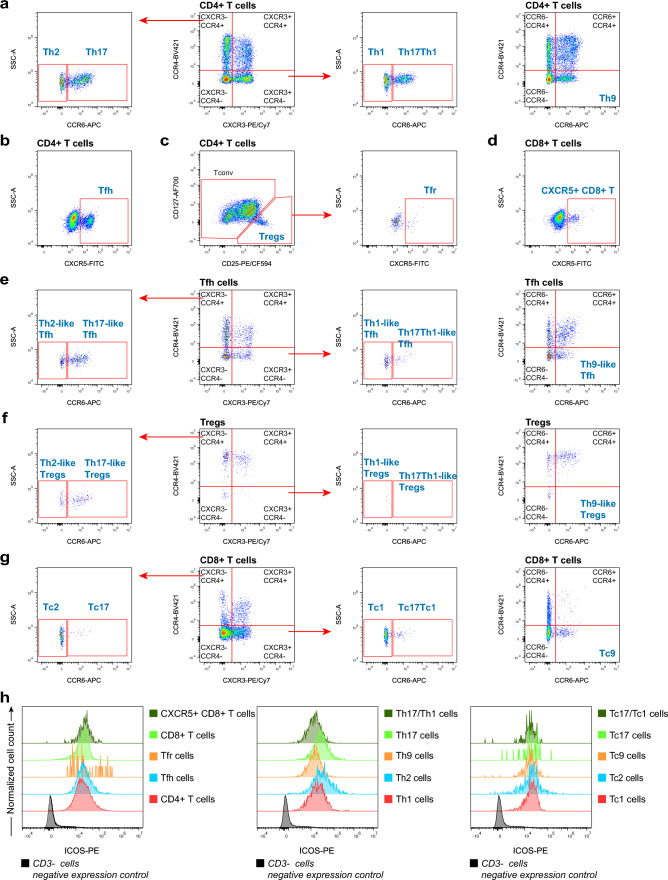
Gating strategies for the Th, Tc, Tfh and Treg cells panel (Panel 5). Exclusion of doublets and gating of CD45^+^ cells as shown for panel 1 (Fig. 2a). CD4^+^ T cells, CD8^+^ T cells, CD4^−^ CD8^−^ T cells, and CD4^+^ CD8^+^ T cells were identified by their expression of CD4 and CD8 (shown in Fig. 4a). **a** Identification of the Th subsets. Th1 (CXCR3^+^ CCR4^−^ CCR6^−^), Th2 (CXCR3^−^ CCR4^+^ CCR6^−^), Th9 (CCR4^−^ CCR6^+^), Th17 (CXCR3^−^ CCR4 + CCR6^+^), and Th17/Th1 (CXCR3^+^ CCR4^−^ CCR6^+^) subsets are identified on CD4^+^ T cells as indicated. **b** Tfh cells were identified on the anti-CXCR5 versus SSC-A plot. **c** CD4^+^ T cells were divided into Tconv cells and Treg cells, which were subsequently distinguished into CXCR5^+^ Tfr cells (anti-CXCR5 versus SSC-A plot). **d** CXCR5^+^ CD8^+^ T cells were gated on the anti-CXCR5 versus SSC-A plot. **e–g** Different Th-like subsets were identified based on the expression of CXCR3, CCR4, and CCR6 for Tfh cells (**e**), Treg cells (**f**), and Tc cells (**g**) with similar gating strategies for Th cells. **h** The expression levels of ICOS of different T cell subsets were evaluated. A reference population of CD3^−^ cells from the same sample was served as a negative expression control

In Panel 5 the CD4^+^ T cells were further distinguished into the Th1 (CXCR3^+^ CCR4^−^ CCR6^−^), Th2 (CXCR3^−^ CCR4^+^ CCR6^−^), Th9 (CCR4^−^ CCR6^+^), Th17 (CXCR3^−^ CCR4 + CCR6^+^), and Th17/Th1 (CXCR3^+^ CCR4^−^ CCR6^+^) (Fig. 6a) (Mahnke et al. 2013b). T follicular helper (Tfh) cells, T follicular regulatory (Tfr) cells, and CXCR5^+^ CD8^+^ T cells were identified by their expression of CXCR5 (Fig. 6b–d). In addition, different Th-like subsets were identified based on the expression of CXCR3, CCR4, and CCR6 for Tfh cells (Fig. 6e), Treg cells (Fig. 6f), and cytotoxic T (Tc) cells (Fig. 6g), with similar gating strategies for Th cells (Brodie et al. 2013; Swieboda et al. 2019; Wingender and Kronenberg 2015). CD278 is a CD28-superfamily costimulatory molecule expressed on activated T cells, also known as inducible T-cell costimulator (ICOS), which was included to further characterize the functional status of individual subsets (Fig. 6h).

#### B Cell Subsets

**Fig. 7:**
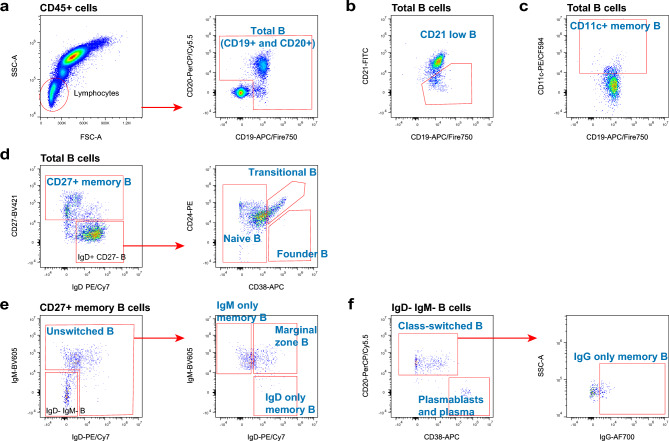
Gating strategies for the B cells panel (Panel 6). Exclusion of doublets and gating of CD45^+^ cells as shown for panel 1 (Fig. 2a). **a** Lymphocytes were gated on the FSC-A versus SSC-A dot plot, and total B cells (CD19^+^ and CD20^+^) were identified on an anti-CD19 versus anti-CD20 dot plot for subsequent analyses. **b** CD21^−/low^ B cells (anti-CD19 versus anti-CD21 plot) were identified on total B cells. **c** CD11c^+^ memory B cells (anti-CD19 versus anti-CD11c plot) were identified on total B cells. **d** The subsets CD27^+^ memory B cells, naïve B cells, transitional B cells, and founder B cells were characterized in a two-step process by their expression of IgD, CD27, CD38, and CD24. **e** CD27^+^ memory B cells were further divided into unswitched B cells and IgD^−^ IgM^−^ B cells. Marginal zone B cells, IgD only memory B cells, and IgM only memory B cells were evaluated on the anti-IgD versus anti-IgM plot. **f** Plasmablasts, plasma cells and class-switched B cells (anti-CD38 versus anti-CD20 plot) were identified on IgD^−^ IgM^−^ B cells. The class-switched B cells were further gated for IgG only memory B cells (anti-IgG versus SSC-A plot)

To identify total B cells, two B cell pan markers CD19 and CD20 were included in panel 6 (Fig. 7a). CD21^−/low^ B cells were identified in Fig. 7b, which represent an innate-like B cell population (Rakhmanov et al. 2009). CD11c^+^ memory B cells were gated on total B cells (Fig. 7c), which are precursors of antibody-secreting cells (Golinski et al. 2020). The CD27^+^ memory B cells, naïve B cells, transitional B cells, and founder B cells were characterized in a two-step process by their expression of IgD, CD27, CD38, and CD24 (Fig. 7d). In addition, we identified unswitched B cells, marginal zone B cells, IgD only memory B cells, and IgM only memory B cells based on their differential expression of IgD and IgM (Fig. 7e). Plasmablasts, plasma cells, and class-switched B cells were identified from IgD^−^ IgM^−^ B cells (Fig. 7f). The gating strategies for B cell subsets presented here refer to the studies of OMIP-003 (Wei et al. 2011), OMIP-047 (Liechti et al. 2018), OMIP-051 (Liechti and Roederer 2019a), and OMIP-068 (Cascino et al. 2020).

## Discussion

Understanding the phenotypes of human immune system is important to define metrics of immunological health. Here, we present a standardized workflow for high-dimensional single-cell immunophenotyping of human whole blood using multi-parametric flow cytometry. Our six optimized panels can also be freely combined with other panels for specific hypothesis-driven studies. The researchers can replicate the procedures easily and avoid costly, time-consuming mistakes during panel development. The robust platform approach of using high-throughput flow cytometry can be applied to biological and medical research.

Our panels enable deep phenotyping of most of the immune cell populations in human peripheral blood. Several groups have designed multicolor flow cytometry panels for the monitoring of immune cells from peripheral blood mononuclear cells (PBMCs) (Moncunill et al. 2014; Nogimori et al. 2021; Park et al. 2020; Payne et al. 2020). However, polymorphonuclear leukocytes such as granulocytes are depleted in PBMCs. Granulocytes are important components of the human innate immune system. The identification of granulocytes including neutrophils, eosinophils and basophils was designed in our assay. Our panels also included the assessment of unconventional T cells (γδ T cells, NKT cells, MAIT cells). Unconventional T cells share functional profiles of both innate and adaptive immunity that play critical roles in many diseases (Bae et al. 2018; Feng et al. 2015; Godfrey et al. 2019, 2018, 2016; Mayassi et al. 2021; Petley et al. 2021; Silva-Santos et al. 2019; Wilkinson and Cerrone 2020). It is important to understand the immune functional status of the unconventional T cells. Furthermore, we identified different Th and Th-like subsets by their expression of CXCR3, CCR4, and CCR6 on classical Th cells, Tfh cells, Tregs, and Tc cells. These designed panels enable us to capture thousands of immune cell traits.

Flow cytometry is a powerful tool that provides rapid multi-parametric analysis of cells at the single-cell level. Additional standardization efforts are also needed in the selection of reagents, instrument setup, sample handling, and data analysis to decrease variability in a longitudinal study or multi-site study (Gratama et al. 2002; Gratama et al. 1997; Kalina 2020; Maecker et al. 2010; Maecker et al. 2012). Several international consortia are developing standardization of flow cytometry protocols and applications such as the EuroFlow Consortium (Kalina et al. 2012; van Dongen et al. 2012), the ONE study consortium (Streitz et al. 2013), the Human Immunology Project Consortium (HIPC) (Brusic et al. 2014; Courtot et al. 2015; Finak et al. 2016; Maecker et al. 2012), the PRECISAIDS project (Jamin et al. 2016), and several other groups (Hasan et al. 2015; Ivison et al. 2018). There are a variety of aims for these projects, and the focuses of standardization differ. These studies provided a reference framework for standardized flow cytometry and inspired our research.

Establishing robust flow cytometry panels requires careful selection of antibody clones and fluorochrome combinations. For researchers starting Panel design, ranking target antigens is a good place to start. Antigen ranking should mainly consider the following three points, including (1) the expression level of the target antigen on the cells of interest, antigens with low expression levels should be given priority; (2) the required resolution of the target antigen, specifically, for functional markers, which usually expression occur along a continuum and therefore need to be considered first, while for lineage markers (e.g. CD3, CD4, etc.), which usually have good separation pattern, can be considered later; and (3) gating strategies, the markers in the back of the gating order should be noted to prevent the influence of the preceding markers. Based on the above antigen ranking, further fluorochromes selection and testing are performed. We conducted extensive antibody testing (we presented a few examples of antibody selection in the Fig. 1) and found significantly different performances of antibody reagents from different suppliers. We suggest choosing suitable antibodies from different suppliers according to the testing results. For instrument setup, we describe the optimized methods based on existing data and experience. Usually, it is subjective to adjust the voltage gains based on the unstained sample. We describe a practical procedure on how to set the optimal voltage gains for each fluorescence detector (Maecker and Trotter 2006; Perfetto et al. 2006, 2012). Furthermore, we noticed the compensation by software automated algorithms needs to be carefully reviewed, and if necessary, adjusted compensation after sample acquisition.

This protocol was optimized for direct staining of human peripheral blood samples which is time-saving and minimizes variations in sample preparation. The procedures for previous studies on immunophenotyping assays were including isolation of PBMCs, freezing and thawing cells. However, several studies have demonstrated that the isolation of PBMCs by Ficoll density gradient centrifugation will alter the expression of cell surface markers, cell subset distribution and function (Appay et al. 2006; Hoffmeister et al. 2003; Maecker et al. 2012, 2005; Renzi and Ginns 1987; Valle et al. 2012). It can also include additional variations owing to freezing/thawing steps. Our immunophenotyping assay was developed for an easy, fast procedure in only 2 mL of human peripheral blood.

Manual gating of flow cytometry data is a major source of variability in flow cytometry analyses. Our gating strategy was designed for batch analysis using FlowJo and minimized the gating adjustment. It is well suited for centralized data analysis (Maecker et al. 2005). Recently the advances in computational flow cytometry make it possible to further explore multiparametric flow cytometry data using high-dimensional analysis methods, such as t-distributed stochastic neighbor embedding (t-SNE) (van der Maaten and Hinton 2008), viSNE (Amir el et al. 2013), Spanning-tree progression analysis of density-normalized events (SPADE) (Qiu et al. 2017), FlowSOM (Van Gassen et al. 2015), FLOWMAP (Zunder et al. 2015), and PhenoGraph (Levine et al. 2015). These computational methods will be more efficient, objective and have better reproducibility (Brummelman et al. 2019; Mair et al. 2016; Saeys et al. 2016). However, there are several challenges that need to be solved such as automated population identification, mapping cell types across samples, etc. (Saeys et al. 2016).

In conclusion, we present six multi-parametric flow cytometry panels for the deep immunophenotyping of human whole blood. The standardized approach and protocols for instrument setup, antibody titration, sample staining, and data quality checks were described. However, this protocol is not designed to investigate the secreted proteins and intracellular proteins of the immune cells such as cytokines, and transcriptional factors. The deep immunophenotyping approach can generate a high informative value of datasets. These huge and high-dimensional data can be analyzed by computational flow cytometry methods. Computational flow cytometry is emerging as an important new field for profiling immunity in humans. It will promote a deeper understanding of the complex heterogeneity of cellular behaviors, individual disease states, and perturbations of human immune system.

### Supplementary Information

Below is the link to the electronic supplementary material.

Supplementary file 1 (PDF 581 KB)

Supplementary file 2 (XLSX 14 KB)

## Data Availability

The datasets generated during and/or analyzed during the current study are available from the corresponding author on reasonable request.

## Abbreviations

AF: Alexa Fluor
APC: Allophycocyanin
BV: Brilliant Violet
CCPs: Negative control compensation particles
CCR: C–C motif chemokine receptor
CD: Cluster of differentiation
CM: Central memory
CXCR: C–X–C motif chemokine receptor
Cy5.5: Cyanine 5.5
Cy7: Cyanine 7
DCs: Dendritic cells
EDTA: Ethylenediaminetetraacetic acid
Eff/mem: Effector/memory
EM: Effector memory
FBS: Fetal bovine serum
FITC: Fluorescein isothiocyanate
FMO: Fluorescence minus one
FSC: Forward scatter
FSC-A: FSC-area
FSC-H: FSC-height
FSC-W: FSC-width
HIPC: Human Immunology Project Consortium
HLA-DR: Human leukocyte antigen-DR isotype
ICOS: Inducible T-cell costimulator
Ig: Immunoglobulin
LIN: Lineage
LLOQ: Lower limit of quantification
LOD: Limits of detection
MAIT cells: Mucosal-associated invariant T cells
mDCs: Myeloid dendritic cells
MFI: Median fluorescence intensity
NK cells: Natural killer cells
NKG2D: Natural killer group 2 member D
NKp46: Natural killer cell p46-related protein
OMIPs: Optimized multicolor immunofluorescence panels
PBMCs: Peripheral blood mononuclear cells
PBS: Phosphate buffered saline
PD-1: Programmed cell death protein 1
pDCs: Plasmacytoid dendritic cells
PE: Phycoerythrin
PerCP: Peridinin–chlorophyll–protein complex
PFA: Paraformaldehyde
PMNs: Polymorphonuclear leukocytes
QC: Quality control
RCPs: Rainbow calibration particles
rCV: Robust coefficient of variance
RT: Room temperature
SBR: Signal-to-background ratio
SE: Spreading error
SI: Stain index
SOM: Self-organizing map
SPADE: Spanning-tree progression analysis of density-normalized events
SSC: Side scatter
SSM: Spillover spreading matrix
Tc cells: Cytotoxic T cells
Tconv cells: Conventional T cells
TCR: T cell receptor
Tfh cells: T follicular helper cells
Tfr cells: T follicular regulatory cells
Th cells: T helper cells
Treg cells: Regulatory T cells
TSCM cells: Stem memory T cells
t-SNE: t-distributed stochastic neighbor embedding
