# Supplementary material for: Deep Immunophenotyping of Human Whole Blood by Standardized Multi-parametric Flow Cytometry Analyses
Source: Phenomics. 2023 Feb 23;3(3):309–28. doi: 10.1007/s43657-022-00092-9 (PMC10260734; doi:10.1007/s43657-022-00092-9)
Supplement: Supplementary file 1 — (PDF 581 KB) [file 43657_2022_92_MOESM1_ESM.pdf]

Supplementary Material

Optional Procedure

Materials and reagents

Fresh human peripheral whole blood was collected in heparin-coated tubes or EDTA anticoagulant tubes. Peripheral blood mononuclear cells (PBMCs) were isolated using Ficoll Paque Plus (Cytiva, Cat#17144003). A list of fluorochrome-conjugated CD4 antibodies used in this procedure can be found in Supplementary Table S2. Following surface staining, cells were washed with FACS buffer. After the final wash, cells were resuspended in Paraformaldehyde (PFA; Sangon Biotech, Cat#A500684-0500). Anti-Mouse Ig, κ/Negative Control Compensation Particles Set (BD, Cat# 552843) were used in the procedures described in this protocol. All samples were acquired on a CytoFLEX LX Flow Cytometer (Beckman Coulter, Cat#C00446) through CytExpert_v2.4 (Beckman Coulter), and analyzed with FlowJo_v10.8.1 (BD). Data plots and statistical analyses were done in FlowJo_v10.8.1 and Microsoft Excel_2016 (Microsoft Corporation).

Reagent setup

1) FACS buffer: PBS (Wisent, Cat#311-010-CL) with 2% Fetal Bovine Serum (FBS; Gibco, Cat#10099141) (vol/vol). FACS buffer can be stored at 2°C to 8°C for up to 2 weeks. Do not freeze.
2) 1% Paraformaldehyde (PFA): Dilute the 4% PFA (Sangon Biotech, Cat#A500684-0500) 1:4 with PBS.

Gain Titration (Voltration) Experiment

1. Thaw cryopreserved PBMCs in a 37°C water bath, and adjust the cell concentration to 10 × 10^6 cells/ml with FACS buffer. In the meantime, prepare 5 mL 12 × 75 mm polypropylene tubes per detector, and label tubes with the name of each detector.
2. Transfer 100 μL of thawed PBMCs into each labelled 5 mL tube.
3. Add 1 μL of a single CD4 antibody labelled with different fluorochrome in each tube (fluorochrome-conjugated CD4 antibodies are presented in **Supplementary Table S2**).
4. Mix thoroughly by vortex, and incubate for 15 min at RT in the dark.
5. Add 2 mL FACS buffer to each tube.
6. Centrifuge the cells at 500× *g* for 5 min at RT.
7. Flick or aspirate to remove supernatant and wash twice.

8. Flick or aspirate to remove supernatant and resuspend in a final volume of 250 µL 1% PFA. Store at 4°C and protect from light and moisture until acquisition. Do not freeze.
9. Check instrument's lasers, mirrors, and filters. Complete daily instrument-specific start-up and quality control (QC) procedures.
10. Adjust the respective detector gain determined with the previous procedure (see subheading Calibration minimal gain procedure, Steps 1-2).
11. Set a gate around the singlet cell population on the FSC-A vs FSC-H dot plot to exclude aggregates.
12. Load one of the CD4 single-stained samples, and acquire 10,000 events.
13. Increase gain setting by 50 V and record 10,000 events for every given detector gain setting.
14. Repeat Step 12-13 for each single-stained sample for each detector. Export data in FCS3.1 file format and load into FlowJo.
15. Calculate the MFI of the positive and the negative population, as well as the rSD for the negative population.
16. Calculate the stain index (SI) for each sample by using the following formula: $\mathrm{SI} = \frac{MFI(pos) - MFI(neg)}{2 \times rSD(neg)}$.
17. For each detector, generate a plot showing the SI as a percentage of maximum SI on the y-axis relative to the gain on the x-axis. Here the choice of gain setting can be selected at 90% of plateau (**Fig. S1d**).
18. Once determined, these gain settings are valid for all samples. Repeat this procedure when a new laser, detector, or filter is installed.

Spillover Spreading Matrix Determination Experiment

1. Vortex the BD Anti-Mouse Ig, κ/Negative Control Compensation Particles Set (BD CompBeads) vigorously. In the meantime, label 5 mL 12 × 75 mm polypropylene tubes for each fluorochrome-conjugated CD4 antibody.
2. Pipette 100 µL of FACS buffer into each tube and then add 20 µL of CompBeads (containing 10 µL positive control beads, along with 10 µL negative control beads) to each tube.
3. Add 1 µL of a single CD4 antibody labelled with different fluorochrome in each tube (fluorochrome-conjugated CD4 antibodies are presented in **Supplementary Table S2**).
4. Mix thoroughly by vortex, and incubate for 15 min at RT in the dark.
5. Add 250 µL FACS buffer to each tube. Store at 2°C to 8°C and protect from light and moisture until acquisition. Do not freeze.
6. Check instrument's lasers, mirrors, and filters. Complete daily instrument-specific start-up and quality control (QC) procedures.

7. Adjust the FSC/SSC gain to place the BD CompBeads on a scale in the light scatter plot and set a gate around the singlet cell population on the FSC-A vs FSC-H dot plot to exclude aggregates.
8. Adjust the detector gain determined with the previous procedure (see subheading Determination of best gain settings).
9. Load one of the CD4 single-stained samples, and acquire 5,000 events per tube.
10. Repeat Step 11 for all single-stained samples. Export data in FCS3.1 file format and load into FlowJo compensation group.
11. Start FlowJo’s compensation wizard, adjust the gate size to contain the representative populations, and move the positive or negative gates in the plot to define the target population.
12. Click the “View Matrix” button (File Band –> View Matrix button), and click the “SSM” button there.
13. Export the spillover spreading matrix (SSM) as a CSV file and color-code the matrix based on SE from no SE (white) to high SE (red).
14. Calculate the row sums and column sums in the SSM. The fluorochrome with the lowest row sum contribute the least spreading error. The detectors with the highest column sums receive more spreading error (**Fig. 1f**).
15. Repeat this procedure when a new laser, detector, or filter is installed.

Supplementary Figure Legends

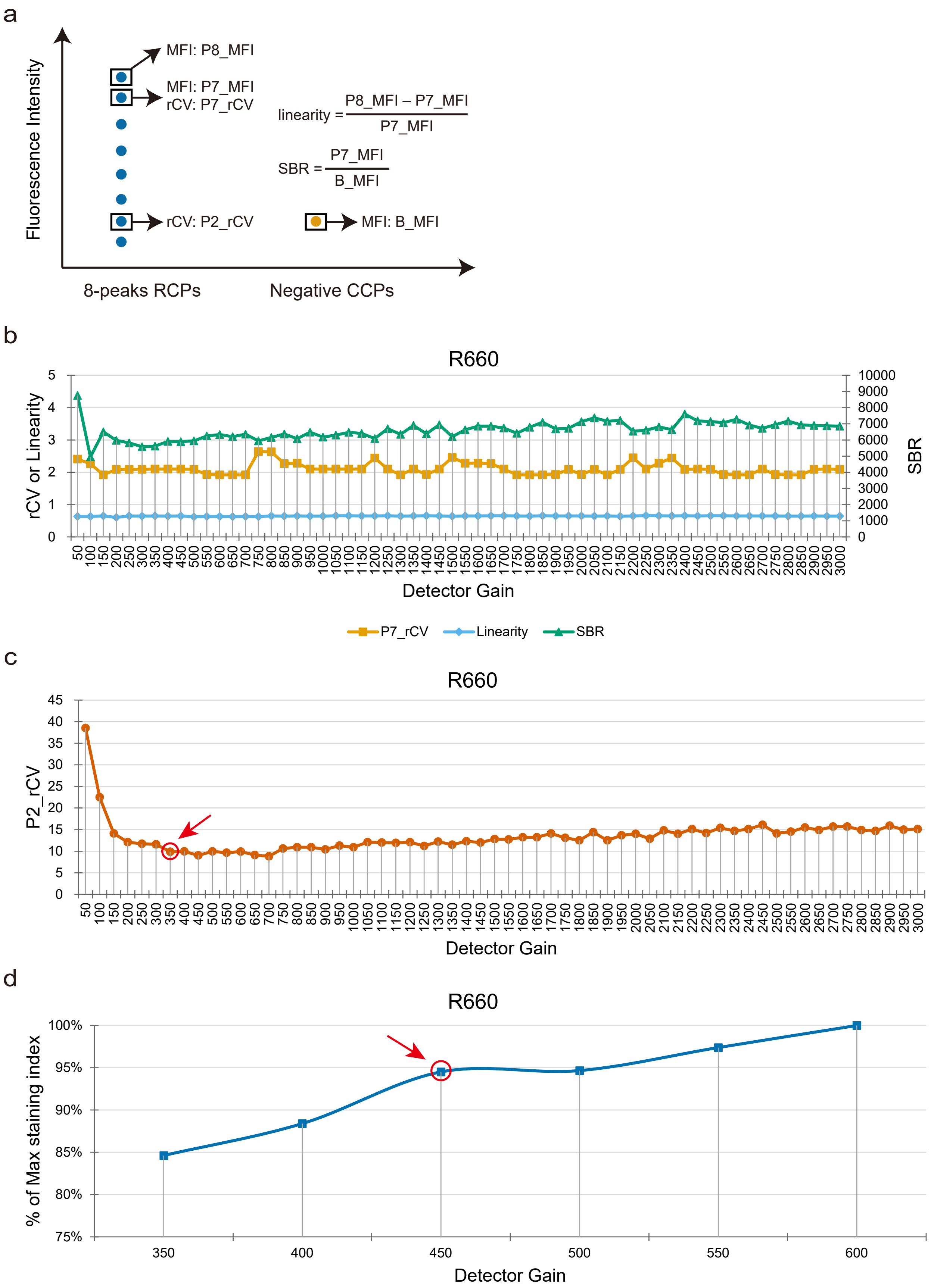

(Legend on next page)

Supplementary Fig. S1 Examples for instrument characterization and detector gain optimization a Collect schematic diagrams of MFI and CV of the 8-peaks RCPs (left) and negative CCPs (right). **b** Illustrate how to plot three curves of the 8-peaks RCPs and negative CCPs collected over a series of gains in the R660 detector. Here, the SBR curves flatten out at 150 V. In this example, the detector gain range can be defined between 150 and 3,000 V. **c** The rCV of the second dimmest peak is plotted in the R660 detector. In this example, the minimum gain is 350 V to bring the negative population out of the electronic noise range of the detector. **d** Examples for voltration of the R660 detector. In this example, the plateau of the stain index is 450 V, indicating that this is the best gain setting to achieve optimal resolution.

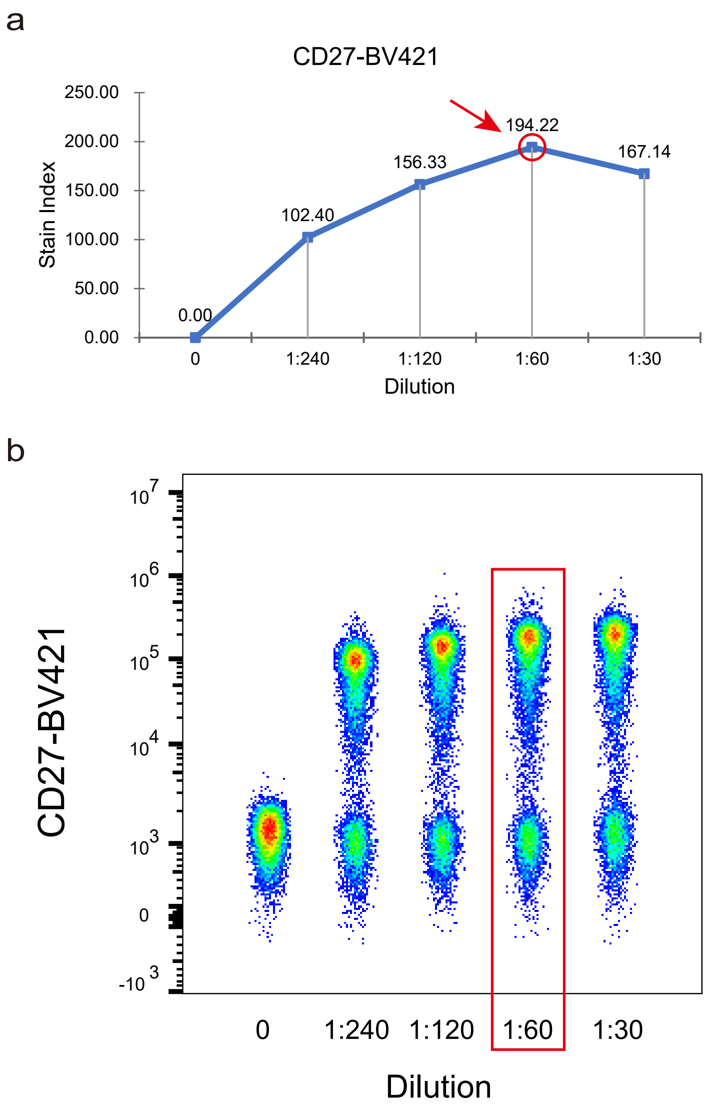

Supplementary Fig. S2 Titrations of BV421 anti-human CD27 antibody a The stain index curve of the BV421 anti-human CD27 antibody. The red arrow indicates that the stain index is highest at the dilution of 1:60. **b** Concatenated plot of titration series for BV421 anti- human CD27 antibody. Selected dilutions are delineated by a red box. Titrations are expressed as μL of antibody in 120 μL of staining volume. The dilution series is 1:30, 1:60, 1:120, 1:240.

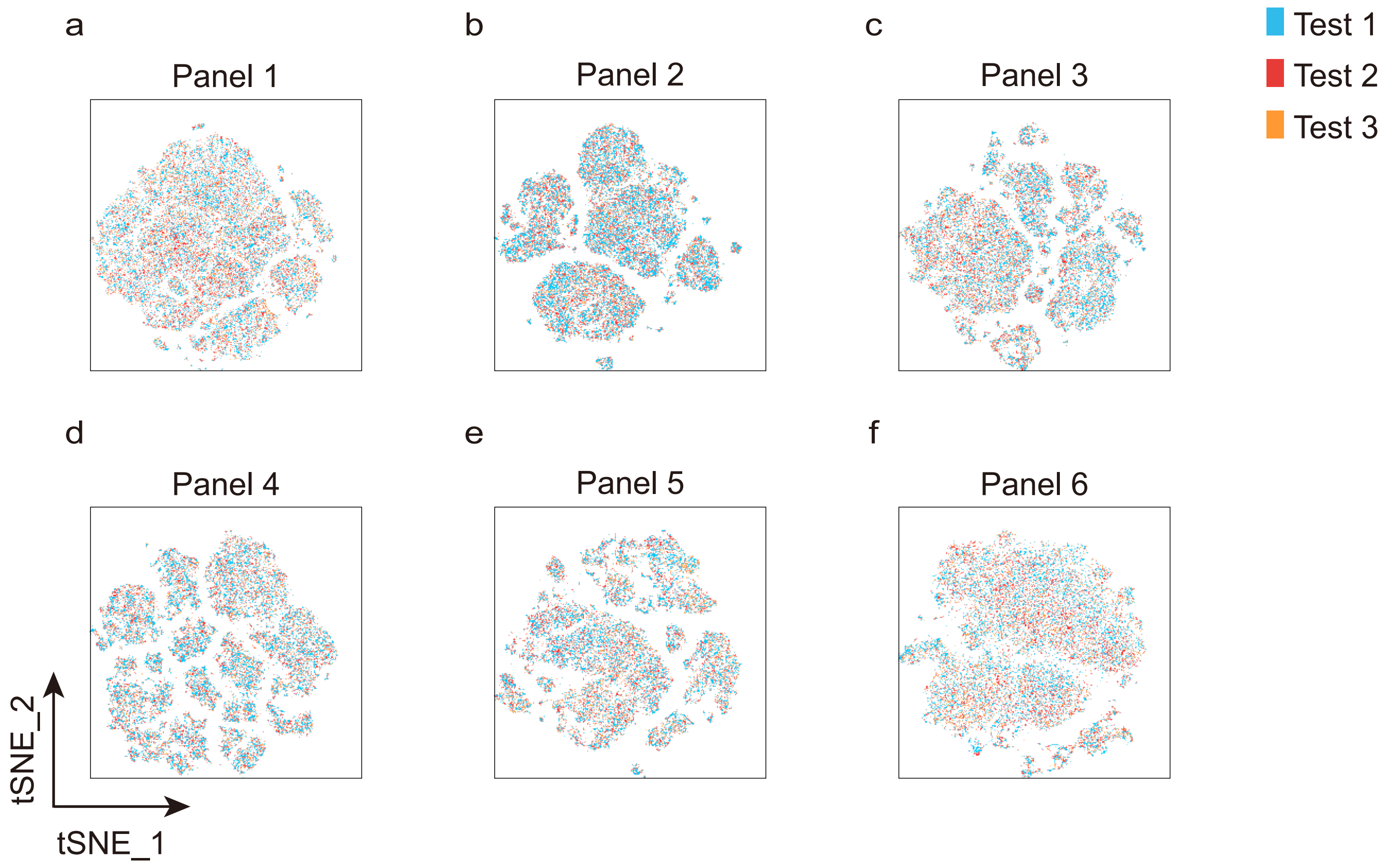

Supplementary Fig. S3 Reproducibility of the human immunophenotyping assay in whole blood Sodium heparin anticoagulant whole blood from healthy individuals was collected. The human immunophenotyping assays were repeated three times from same samples. **a-f** Data shown use t-distributed stochastic neighbor embedding (t-SNE) for dimensionality reduction for Panel 1 to Panel 6. The t-SNE plots with cells colored according to different tests. Data are representative of results from at least three independent experiments.
